# Effects of an Extreme Weather Event on Primate Populations

**DOI:** 10.1002/ajpa.25049

**Published:** 2025-01-06

**Authors:** Megan Beardmore‐Herd, Meredith S. Palmer, Kaitlyn M. Gaynor, Susana Carvalho

**Affiliations:** ^1^ Primate Models for Behavioural Evolution Lab, Institute of Human Sciences University of Oxford Oxford UK; ^2^ Interdisciplinary Centre for Archaeology and the Evolution of Human Behaviour (ICArEHB) Universidade do Algarve Faro Portugal; ^3^ Center for Biodiversity and Global Change Yale University New Haven Connecticut USA; ^4^ Department of Zoology and Botany University of British Columbia Vancouver British Columbia Canada; ^5^ Department of Science Gorongosa National Park Sofala Mozambique; ^6^ CIBIO/InBIO, Centro de Investigação em Biodiversidade e Recursos Genéticos, Universidade do Porto Campus de Vairão Vairão Portugal

**Keywords:** baboons, camera traps, cyclone, ecological pressure, vervet monkeys, armadilhas fotográficas, babuínos, ciclone, macacos vervet, pressão ecológica

## Abstract

**Objectives:**

With contemporary, human‐induced climate change at a crisis point, extreme weather events (e.g., cyclones, heatwaves, floods) are becoming more frequent, intense, and difficult to predict. These events can wreak rapid and significant changes on ecosystems; thus, it is imperative to understand how wildlife communities respond to these disruptions. Primates are perceived as being a largely adaptable order, but we often lack the quantitative data to rigorously assess how they are impacted by extreme environmental change. Leveraging detections from a long‐term camera trap survey, this opportunistic study reports the effects of an extreme weather event on a little‐studied population of free‐ranging primates in Gorongosa National Park, Mozambique.

**Materials and Methods:**

We examined shifts in gray‐footed chacma baboon ( *Papio ursinus griseipes* ) and vervet monkey ( *Chlorocebus pygerythrus* ) spatial distribution and relative abundance following Cyclone Idai—a category four tropical cyclone that struck Mozambique in March 2019.

**Results:**

Baboon spatial distributions were impacted in the first month after the cyclone, with more detections in areas where flooding was less severe. Spatial distributions renormalized once floodwaters began to recede. We describe vervet monkey spatial distribution trends, though sample size limitations inhibited statistical analysis. Primate relative abundance did not appear to substantially decrease following the cyclone, suggesting troops were able to adopt behavioral adjustments to evade rising floodwaters.

**Discussion:**

These findings highlight the behavioral flexibility of Gorongosa's primates and their ability to adapt to extreme—if temporary—disruptions, with implications for primate conservation in the Anthropocene and research into how rapid climatic events may have shaped primate evolution.

## Abstract (pt)

**Efeitos de um Evento Climático Extremo sobre as Populações de Primatas**

**Resumo:**

**Objetivos**

Com as mudanças climáticas contemporâneas induzidas pelo ser humano em um ponto de crise, eventos climáticos extremos (por exemplo, ciclones, ondas de calor, inundações) estão se tornando mais frequentes, intensos e difíceis de prever. Esses eventos podem causar mudanças rápidas e significativas nos ecossistemas; portanto, é crucial entender como as comunidades de vida selvagem respondem a essas perturbações. Os primatas são vistos como uma ordem amplamente adaptável, mas frequentemente carecemos de dados quantitativos para avaliar rigorosamente seu impacto por mudanças ambientais extremas. Aproveitando detecções de um levantamento de armadilhas fotográficas de longo prazo, este estudo oportunista relata os efeitos de um evento climático extremo sobre uma população pouco estudada de primatas de vida livre no Parque Nacional da Gorongosa, Moçambique.

**Materiais e Métodos**

Examinamos mudanças na distribuição espacial e na abundância relativa de babuínos de pés cinzentos ( *Papio ursinus griseipes* ) e macacos vervet ( *Chlorocebus pygerythrus* ) após o Ciclone Idai—um ciclone tropical de categoria quatro que atingiu Moçambique em março de 2019.

**Resultados**

As distribuições espaciais dos babuínos foram afetadas no primeiro mês após o ciclone, com mais detecções em áreas onde as inundações foram menos severas. As distribuições espaciais se normalizaram quando as águas das inundações começaram a recuar. Descrevemos as tendências na distribuição espacial dos macacos verdes, embora limitações no tamanho da amostra tenham impedido análises estatísticas. A abundância relativa dos primatas não pareceu diminuir substancialmente após o ciclone, sugerindo que os grupos foram capazes de adotar ajustes comportamentais para evitar as águas das inundações em elevação.

**Discussão**

Esses achados destacam a flexibilidade comportamental dos primatas de Gorongosa e sua capacidade de se adaptar a perturbações extremas—embora temporárias—com implicações para a conservação dos primatas no Antropoceno e para a pesquisa sobre como eventos climáticos rápidos podem ter moldado a evolução dos primatas.

## 1 Introduction

Extreme weather events are expected to become more frequent, intense, and difficult to accurately predict as the climate crisis escalates largely in response to anthropogenic emissions of greenhouse gases (Balaguru et al. 2016; Bhatia et al. 2018; Emanuel 2005, 2017; Sobel et al. 2016). Nonhuman primates (herein, primates) are ecologically and anthropologically significant, but, with their long life histories and slow reproductive rates, are acutely vulnerable to the effects of climate change and extreme weather events (Bernard and Marshall 2020; Jones 2011). Furthermore, among the mammalian orders, primates have experienced a disproportionate level of exposure to cyclones in recent years, exhibiting the highest degree of spatial overlap with geographic areas affected by cyclones recorded between 1992 and 2005 (Ameca y Juárez et al. 2013). However, our understanding of primate responses to extreme climatic events such as cyclone remains limited owing to the low incidence of comparative before and after data on either side of these largely unpredictable occurrences. Studies have shown that extreme weather events can have major and varied impacts on mortality, fertility, social organization and affiliation, home range use, and even gene expression among primate populations (Behie and Pavelka 2005; Johnson et al. 2011; Morcillo et al. 2020; Pavelka et al. 2003; Pavelka, McGoogan, and Steffens 2007; Schaffner et al. 2012; Testard et al. 2021; Watowich et al. 2022). It is imperative that we gain a deeper awareness of how primates respond to extreme weather events, both so we may better support present primate populations through improved and targeted wildlife conservation practices, and so we may further our understanding of past primates through research into how major climatic events may have shaped early primate and human evolution.

Already the impacts of the climate crisis are being felt in Mozambique, with extreme weather events in the form of cyclones, severe floods, droughts, and wildfires occurring frequently in this southern African country and projected to be exacerbated as global temperatures rise (Hoffmann et al. 2009; Hope 2019; Mavume et al. 2021; Post‐Cyclone Idai Cabinet for Reconstruction 2019). The first half of 2019 witnessed especially devastating weather, with the incidence of the strongest recorded tropical cyclone in the Southern Hemisphere to date: Cyclone Idai. On the night of March 14, 2019, Cyclone Idai, a category four tropical cyclone, made landfall near the port city of Beira in central Mozambique. Cyclone Idai brought high winds (up to 220 km/h) and heavy rainfall (> 200 mm in 24 h), leading to severe, widespread flooding and entire communities being submerged under 10 m of floodwater (Charrua et al. 2021; Devi 2019; Post‐Cyclone Idai Cabinet for Reconstruction 2019). The aftermath of Cyclone Idai was catastrophic, affecting over 1.5 million Mozambicans, claiming the lives of over 600 people, and displacing hundreds of thousands more (Post‐Cyclone Idai Cabinet for Reconstruction 2019).

**FIGURE 1:**
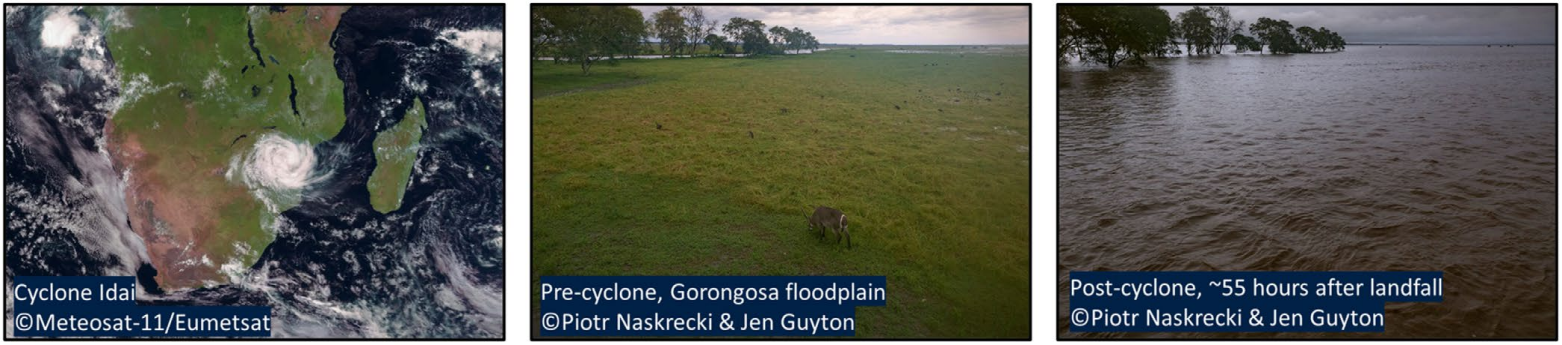
Map showing Cyclone Idai over Mozambique, as captured by Meteosat‐11 (copyright: EUMETSAT) at 9:15 a.m. UTC on March 15, 2019 (left), and two photographs taken by Piotr Naskrecki and Jen Guyton of the Gorongosa National Park floodplain from the same location showing the severity of the flooding that the cyclone brought to the park, the first being taken on March 15, 2019 approximately 18 h after Cyclone Idai made landfall in Mozambique but before it reached the park (center), and the second taken on March 17, 2019 approximately 55 h after landfall (right).

Cyclone Idai not only devastated human communities but also had significant impacts on Mozambique's wildlife. This was particularly apparent in Gorongosa National Park, which experienced severe flooding centered around its main water body, Lake Urema (Figure 1). Gorongosa National Park and the surrounding areas regularly experience seasonal flooding and drought periods, and there is evidence that this region may have been a cyclone hotspot during the early and middle Miocene (Yan et al. 2019). However, as highlighted above, Cyclone Idai was especially severe (Vittaz 2021). Understanding its impact on the park's animal communities will contribute towards the implementation of effective mitigation practices in response to future extreme weather events.

When Cyclone Idai struck, ongoing studies of the large herbivore and carnivore species in Gorongosa National Park enabled researchers to combine multiple data sources to collaboratively investigate the sensitivity of resident large mammals to the cyclone across timescales of hours, days, and months (Walker et al. 2023). Data from camera traps, GPS collars, aerial surveys, and DNA metabarcoding suggested that many herbivores moved to higher ground and shifted their diets in response to rising floodwaters. Smaller‐bodied herbivores and those strongly affiliated with lowland habitats were disproportionately affected by Cyclone Idai, with populations of these species falling in the months following the cyclone. However, for larger‐bodied and more upland‐affiliated herbivores, population size increased on average. These findings align with predictions that larger mammals cope better with these types of disasters, as smaller mammals are limited in their mobility and therefore less able to escape rising floodwaters and more sensitive to the food quantity and quality declines they cause. Whether these predictions hold for comparatively small‐bodied primates, known for their behavioral flexibility and ability to exploit both terrestrial and arboreal niches, warrants further attention.

At the time of Cyclone Idai, primatology research had only just begun in Gorongosa National Park (Hammond et al. 2022; Lewis‐Bevan et al. 2020). In May 2018, Lewis‐Bevan et al. (2020) began to habituate and study the behavior of a troop of gray‐footed chacma baboons ( *Papio ursinus griseipes* ) that range on the floodplain of Gorongosa National Park (a troop whose core habitat experiences flooding on an annual basis) and was able to perform an opportunistic analysis of survivorship and group demography following Cyclone Idai. Minimal demographic changes in the population were observed in the months following the cyclone; while a number of infants and juveniles went missing, there were no recorded instances of adult mortality among this troop of baboons (Lewis‐Bevan et al. 2020). This case study suggests that younger individuals were more vulnerable, as the adult's previous, regular exposure to high levels of seasonal flooding may have helped them mitigate the negative impacts of Cyclone Idai through behavioral adjustments including temporary habitat shifts.

Here, we set out to investigate the behavioral effects of Cyclone Idai on primates in Gorongosa National Park at the population level, building on these troop‐level findings for baboons and complementing the multi‐level investigations on large mammals. We used the camera trap survey from Walker et al. (2023) to document and analyze cyclone impacts on the spatial distribution and relative abundance of two primate species in Gorongosa National Park: gray‐footed chacma baboons and vervet monkeys ( *Chlorocebus pygerythrus* ).

We compared primate spatial distributions and relative abundance 1 month before Cyclone Idai and in the first 7 months that followed to matched time intervals from the previous two non‐cyclone‐affected years (2017 and 2018) to control for seasonal effects. We assessed how primate detection rates varied with flood severity and flood level retreat over time. We hypothesized that there would be an interaction between cyclone incidence, flood severity, and flood level retreat over time, such that, in the months immediately after Cyclone Idai, there would be fewer primates detected at camera trap sites where flood severity was highest, as compared to matched months in years without cyclone activity. We anticipated that this effect would be most pronounced in the first month immediately following the cyclone, with spatial distributions gradually renormalizing over the following months. Such a pattern would reflect the behavioral flexibility of these primates, providing insights into the ways that ecological pressures may have shaped primate evolution and how present primate populations may respond to rapid ecological change in the Anthropocene.

## 2 Materials and Methods

### 2.1 Study Site and Species

Gorongosa National Park is a 4067 km^2^ protected area situated at the southernmost end of the Great Rift Valley in central Mozambique, southeast Africa (Correia et al. 2017). The park covers a mosaic of ecosystems, including closed‐canopy forests, savannas, and floodplain grasslands (Daskin, Stalmans, and Pringle 2016), with as many as 15 broad types of landscape recognized by their unique combinations of vegetation and rainfall (Stalmans and Beilfuss 2008). The climate is highly seasonal, with peak rainfall occurring during the wet season (December—March) known to cause up to 40% of Gorongosa National Park to flood. This is followed by a harsh dry season (April—November) characterized by wildfires throughout the savanna and grassland areas and the progressive drying out of most water pans and rivers (Daskin, Stalmans, and Pringle 2016; Stalmans et al. 2019; Tinley 1977). In the center of Gorongosa National Park lies Lake Urema, one of the park's only permanent water bodies, upon which the entire ecosystem relies (Chirindja et al. 2016).

Prior to the incidence of Cyclone Idai, the park's animal communities faced disruptions of another kind which they are still in a state of recovery from. Once a biodiversity hotspot, the fauna of Gorongosa National Park was drastically reduced during the Mozambican Civil War (1977–1992), with large mammal populations suffering declines in excess of 90% as a result of the conflict (Hatton, Couto, and Oglethorpe 2001; Stalmans et al. 2019). Efforts led by the Gorongosa Restoration Project, which works with local communities and scientists to conserve and restore wildlife, have resulted in recovery among most populations, with some mesoherbivore species even surpassing pre‐war population levels (Stalmans 2012; Stalmans, Peel, and Massad 2014; Stalmans, Peel, and Gonçalves 2018; Stalmans et al. 2019; Stalmans and Peel 2016).

Ongoing research as part of the Paleo‐Primate Project Gorongosa is beginning to uncover information about Gorongosa's previously unstudied primate populations. Initial studies have focused on Gorongosa's baboons, exploring their phenostructure, evolutionary adaptation, and behavior (Baehren and Carvalho 2022; Bobe, Martinez, and Carvalho 2020; Hammond et al. 2022; Martinez et al. 2019; Muschinski et al. 2020; Muschinski, Mielke, and Carvalho 2023; Santander et al. 2022). Biennial aerial wildlife counts and the ongoing camera trap survey have helped assess the size of the baboon population in Gorongosa National Park (Gaynor et al. 2021; Stalmans, Peel, and Massad 2014; Stalmans, Peel, and Gonçalves 2018, 2022; Stalmans and Peel 2016, 2020). During the 2016 aerial wildlife count, 202 baboon troops were recorded across an 1832 km^2^ count block which represents 49.7% of Gorongosa National Park, followed by 193 baboon troops in 2018, and 190 in 2020 (M. Stalmans, personal communication, April 13, 2023). Individual baboon numbers were not enumerated during the aerial wildlife counts. Thus, while a valuable indicator of the baboon population in Gorongosa, the number of baboon troops recorded in the aerial wildlife counts is subject to group fission and fusion events, troop dispersals into and out of the count block, and the accuracy with which separate baboon troops can be detected from the air, meaning caution should be practiced when using the troop counts to make inferences about the total baboon population size or growth rates across time.

While vervet monkeys are frequently sighted in Gorongosa National Park, there are currently no estimates of the number of troops in the area as their small size precludes them from being included in the aerial wildlife counts. Vervet monkeys are however estimated to be less widespread than baboons based on a study using the first season of data from the Gorongosa National Park camera trap survey (Gaynor et al. 2021). This study found baboons to be widespread across all surveyed areas and predicted that this may be linked to reduced predator presence in Gorongosa National Park following the Mozambican Civil War (Gaynor et al. 2021).

### 2.2 Data Collection

**FIGURE 2:**
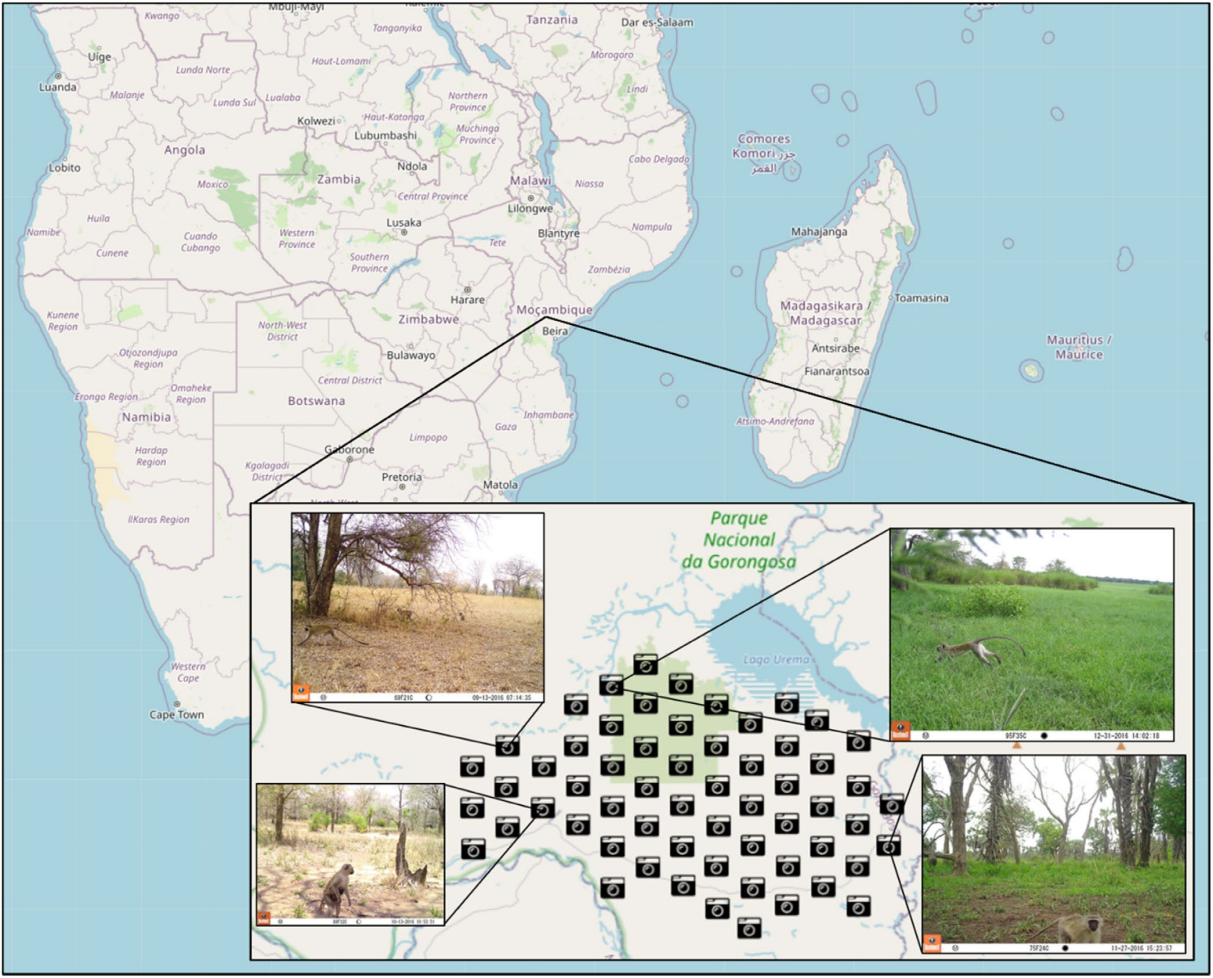
Map of Southern Africa showing the location of Gorongosa National Park, inset with a close‐up showing the location of camera sites within the Gorongosa National Park camera trap survey in relation to Lake Urema, which is further inset by images of vervet monkeys from four of the camera sites representing the diversity of habitats sampled and the raw images that the primate detection data are obtained from.

The present study used data from the Gorongosa National Park camera trap survey, which was established in 2016 to evaluate spatiotemporal patterns of mammal activity in Gorongosa National Park (Gaynor et al. 2021). The camera trap survey consists of ~60 cameras distributed systematically over a 300 km^2^ area of savanna woodland located south of Lake Urema within Gorongosa National Park (Figure 2). This region was selected due to its accessibility and safety and because it was known to contain a high density of mammals.

KM Gaynor divided the study area into 5 km^2^ hexagonal grid cells and placed one Bushnell TrophyCam camera approximately in the center of each, mounted on a tree at a height of 1 m and angled downwards. Where possible, cameras were set up facing clearings or trails showing signs of animal activity to increase the likelihood of mammal detections. KM Gaynor collected and classified the 2016–2018 data, documenting details such as the species present, count of individuals in each image, and behavior being performed (as described in Gaynor et al. 2021). MS Palmer led the collection of the 2018–2019 data, which were classified by volunteers on WildCam Gorongosa. For more details on WildCam Gorongosa, see https://www.wildcamgorongosa.org.

For this study, detection data classified as containing baboons or vervet monkeys between June 26, 2016 and October 11, 2019 were downloaded and subset for analysis.

### 2.3 Data Analysis

We aimed to compare the occurrences of baboons and vervet monkeys across the Gorongosa National Park camera trap survey in the year of the cyclone (2019) and the previous two non‐cyclone years (2017 and 2018), focusing on the time intervals corresponding to the month before Cyclone Idai made landfall and the seven subsequent months after the cyclone. Based on the results of Walker et al. (2023) that the spatial distribution of most species renormalized before the end of this period, detections occurring outside of these intervals were not considered in our study. We subset detection data for each year into 30‐day‐long intervals representing the month relative to the date Cyclone Idai reached the park (March 15, 2019). Intervals of this length were used to balance robust and meaningful trend detection over time with reduced sensitivity to short‐term random fluctuations. The interval corresponding to the month before Cyclone Idai (February 13 to March 14) was classified as month − 1, the interval corresponding to the first month after Cyclone Idai (March 16 to April 14) as Month 1, and so on up until the interval corresponding to the seventh month after Cyclone Idai (September 12 to October 11).

**FIGURE 3:**
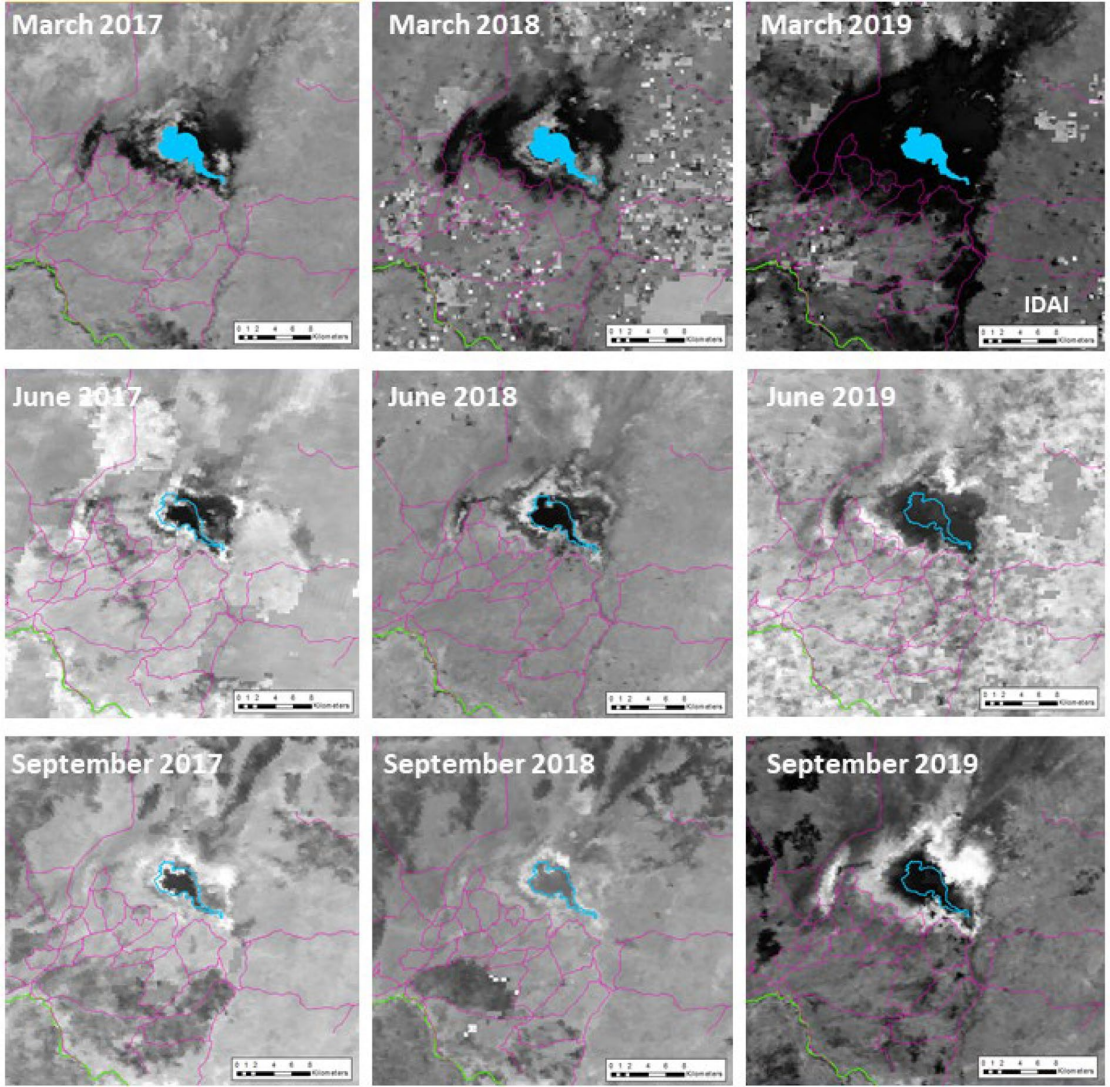
Visual illustration of the extent of Lake Urema and its expansion across the floodplain from March 2017 to September 2019, using the MODIS MOD09Q1 product, reprinted with permission from Stalmans and Peel (2020). The blue extent represents the position of Lake Urema and the pink lines show the location of roads. Note the extreme flooding centered around Lake Urema following Cyclone Idai in March 2019 and the much larger lake area throughout the dry season compared to the previous two non‐cyclone years.

Our goal was to determine whether cyclone‐year distributions were notably different to non‐cyclone‐year distributions and whether this effect was modulated by flood severity and flood level retreat over time. Following Walker et al. (2023) whose analysis of flood sensor data in Gorongosa National Park found that flood depth postcyclone exhibited an exponential relationship with distance from Lake Urema, we used the logarithm of the distance from Lake Urema as a proxy for flood severity. This choice was further supported by patterns of seasonal flooding seen during non‐cyclone years (Figure 3). The month relative to cyclone landfall was used as a proxy for flood‐level retreat.

To reduce sampling bias, we included only ‘independent’ baboon and vervet monkey detections in our datasets. For the present study, detections were considered to be independent if occurring at different cameras or separated by 15 min at the same camera. The removal of non‐independent detections was carried out to reduce the risk of artificially inflated abundance estimates which may be created by individuals of the same troop triggering the camera multiple times in a short period (O'Connell, Nichols, and Karanth 2011). Our definition of independence is consistent with that used to monitor the recovery of the wildlife of Gorongosa National Park using the same camera trap survey (Gaynor et al. 2021; Walker et al. 2023), thereby increasing the comparability of our findings.

To account for variation in sampling effort, we assessed the operation of all cameras across the Gorongosa National Park camera trap grid. This involved accounting for periods when individual cameras were inactive due to technical malfunctions or unable to accurately capture passing wildlife as a result of environmental interference, such as vegetation obscuring a camera's field of view (as per Gaynor et al. 2021).

Detections of baboons and vervet monkeys were described and analyzed separately. We deemed datasets sufficient for inclusion in statistical analysis if there were ≥ 10 independent detections in each 30‐day‐long interval for 2017, 2018, and 2019. While this was the case for baboons, there were < 10 independent vervet monkey detections recorded in five of the 30‐day‐long intervals (corresponding to month − 1 in 2018 and 2019, Month 1 in 2018 and 2019, and Month 2 in 2019). Thus, it was not possible to model the effect of flood severity and flood level retreat on vervet monkey detections. We instead examined qualitative trends in the vervet monkey data.

We used a generalized linear mixed model (GLMM) to examine the relationship between the distribution of baboon detections (frequency relative to monthly average) across the Gorongosa National Park camera trap survey and the level of site‐specific flood severity, and how this relationship changed as time elapsed and floodwaters receded (Zuur, Ieno, and Meesters 2009). The frequency of independent baboon detections per month at each camera was used as the response variable in the GLMM. The month and the logarithm of the distance to Lake Urema were used as fixed effects. The camera trap site was used as a random effect. To control for differences in camera sampling effort, the total number of days per 30‐day‐long interval that each camera was operational was used as an offset in the model (Wearn and Glover‐Kapfer 2017). The total number of independent baboon detections recorded across all cameras in the Gorongosa National Park camera trap survey for each month analyzed was also used as an offset, effectively accounting for annual changes in activity, and thus detectability, which may have been due to seasonal patterns or interannual changes in population density. Lastly, the GLMM used a negative binomial distribution as it is suitable for right‐skewed count data (Zuur, Ieno, and Meesters 2009).

Finally, to explore whether the cyclone affected primate relative abundance, we calculated the number of detections per camera trap day (an index of relative abundance) for each primate species, at each camera, during each month (O'Brien 2011; O'Brien, Kinnaird, and Wibisono 2003). Values for each condition were calculated by dividing the total number of detections by the number of camera trap days (based on dates of camera deployment and operation). Values were only calculated if the condition had ≥ 10 days of camera operation. If a camera had < 10 operation days in a given month, we considered the sampling effort insufficient for calculation of a reliable indicator of relative abundance for that condition.

All data cleaning, visualization, and analysis were conducted in R Studio using the R programming language version 4.1.2 (R Core Team 2021).

## 3 Results

A total of 12,448 independent baboon detections were recorded between February 13 and October 11 across the three study years which represented a total of 23,832 camera trap days across 55 cameras. Baboons were detected at all 55 cameras across this period.

There were substantially fewer detections overall for vervet monkeys (684 independent detections throughout the 3‐year study period), as compared to baboons. During these times, vervet monkeys were detected at 44 of the 55 cameras in the survey.

### 3.1 Spatial Distribution Shifts After Cyclone Idai

**FIGURE 4:**
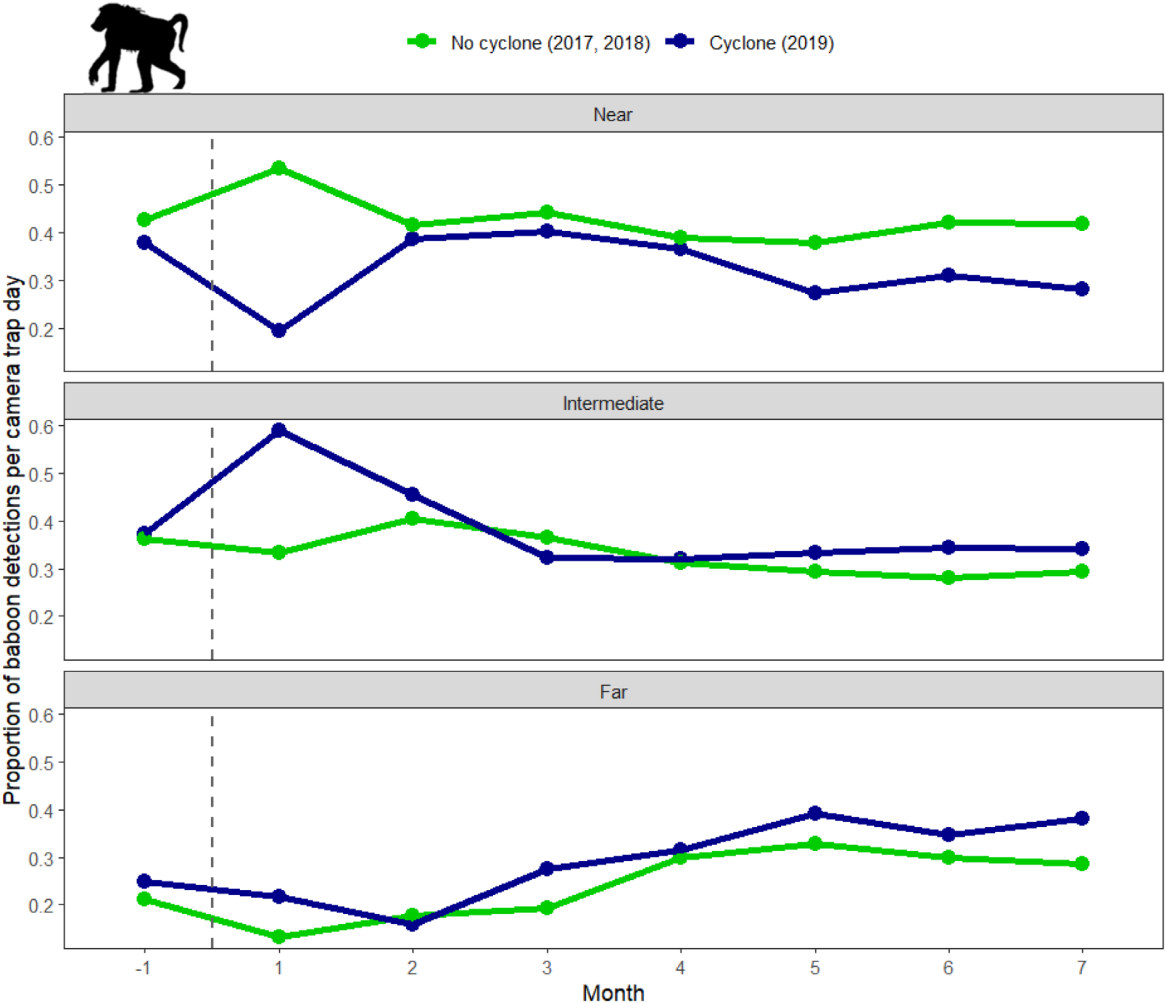
Proportion of baboon detections per camera trap day at cameras classified as near, intermediate, and far from Lake Urema, shown across time in the year of the cyclone (2019) and two non‐cyclone years (2017 and 2018). The cameras are classified based on their distance to Lake Urema for visualization purposes only (following classifications described in Walker et al. 2023), the continuous logarithm of distance to Lake Urema was used as a fixed effect for statistical modeling. The dashed vertical line represents the date when Cyclone Idai made landfall in Gorongosa National Park (March 15, 2019). Fluctuations in the slope of the blue line show how Cyclone Idai displaced baboons from areas near Lake Urema where flooding was most severe, but only during the first month after cyclone incidence.

For baboons, there was a shift in the spatial distribution of detections after the incidence of Cyclone Idai consistent with baboons moving away from Lake Urema and into less flooded areas (Figure 4). By the second month after the cyclone, spatial distribution patterns were once again similar to those in non‐cyclone years.

**FIGURE 5:**
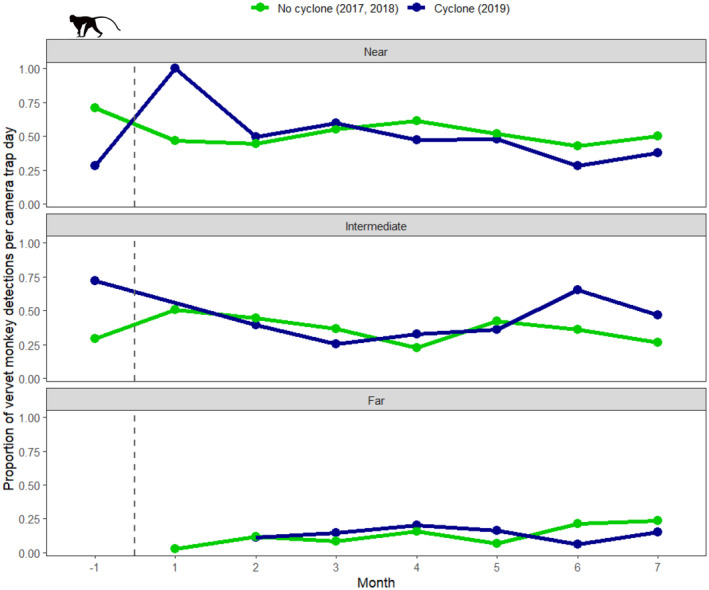
Proportion of vervet monkey detections per camera trap day at cameras classified as near, intermediate, and far from Lake Urema, shown across time in the year of the cyclone (2019) and two non‐cyclone years (2017 and 2018). The cameras are classified based on their distance to Lake Urema for visualization purposes only (following classifications described in Walker et al. 2023), the continuous logarithm of distance to Lake Urema was used as a fixed effect for statistical modeling. The dashed vertical line represents the date when Cyclone Idai made landfall in Gorongosa National Park (March 15, 2019). Note that for some bins, no vervet monkey detections were recorded. Complete data are present from the second month onwards, and here, the slopes of the two lines are similar, suggesting that vervet monkey distributions had been renormalized by the second‐month post‐cyclone.

For vervet monkeys, the low incidence of detections made it difficult to assess spatial distribution shifts in the first month after Cyclone Idai. In this month, vervet monkeys were not detected by any cameras classified as intermediate or far from Lake Urema, thus making it appear as though vervet monkeys shifted their use of space to areas where flooding was most severe (Figure 5). From the second month after Cyclone Idai however, vervet monkey spatial distribution patterns were mostly comparable to those recorded in non‐cyclone years.

### 3.2 The Effect of Cyclone Incidence, Flood Severity, and Time on Detections

**FIGURE 6:**
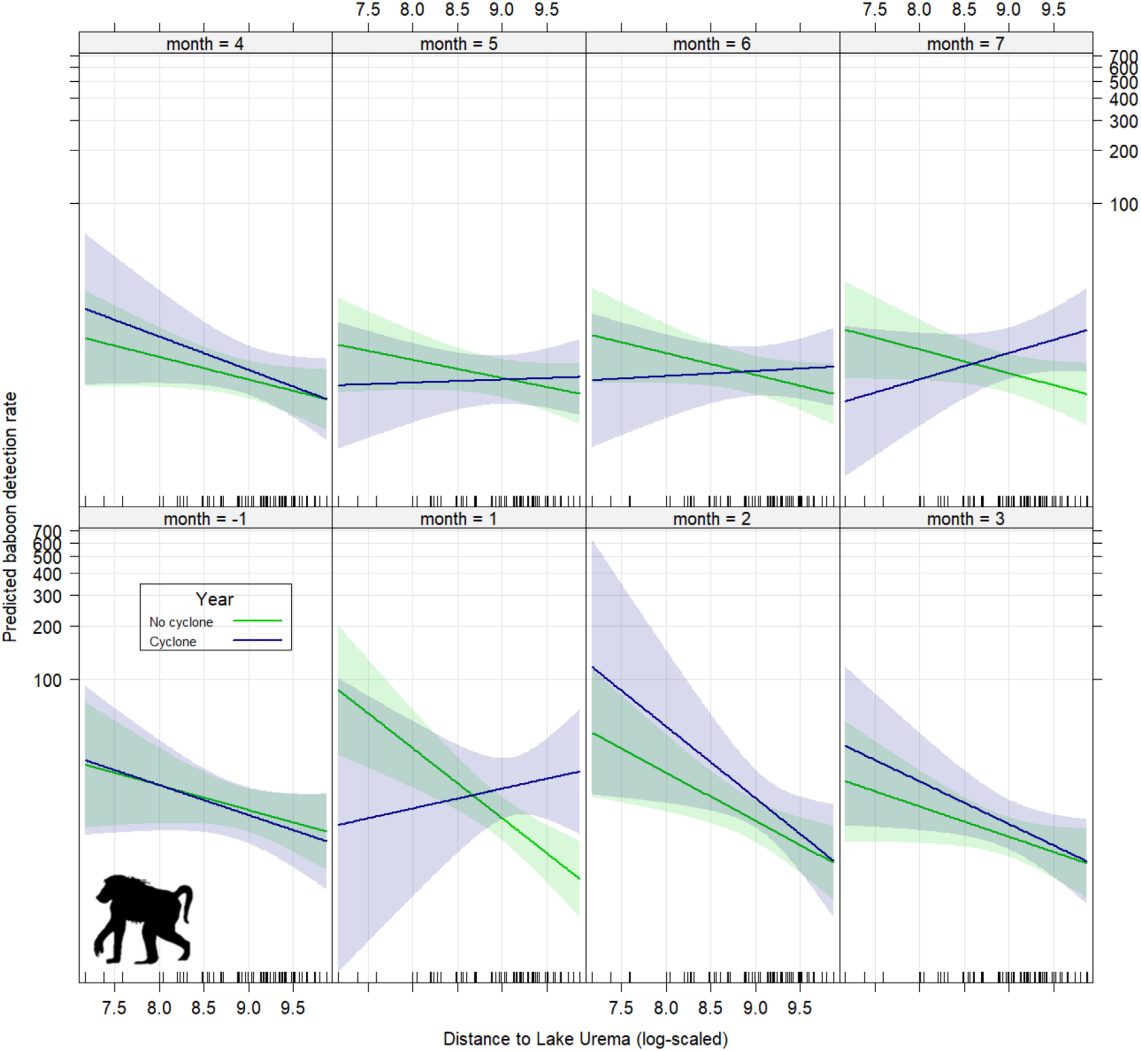
Effects plots showing predicted monthly baboon detection rates as a function of distance from Lake Urema for the year of the cyclone (2019) and two non‐cyclone years (2017 and 2018). In month − 1, before the incidence of Cyclone Idai, the spatial distribution of baboons is the same in the year of the cyclone as in non‐cyclone years, with a downward slope representing higher predicted baboon detection rates in areas closer to Lake Urema. In Month 1, after the incidence of the cyclone, this pattern reverses, before renormalizing by Month 2.

During the period before Cyclone Idai made landfall (month − 1), we observed comparable baboon distribution patterns in both the cyclone year and matched time interval in non‐cyclone years, with higher baboon detection rates at sites closer to Lake Urema (Figure 6). However, during the first month following Cyclone Idai's landfall in 2019 (Month 1), the opposite pattern emerged, with higher baboon detection rates at sites further from Lake Urema (i.e., where post‐cyclone flooding was less severe). We found a significant three‐way interaction between cyclone incidence, distance to Lake Urema, and month during this period (Cyclone year × Lake distance × Month 1: estimate = 1.25; SE = 0.58; *p*‐value = 0.03). This suggests that Cyclone Idai altered the distribution of baboons relative to Lake Urema during the first month after landfall relative to non‐cyclone years and pre‐cyclone distribution patterns. Similarly, we found significant effects for the interval representing Month 1 (Month 1: estimate = 5.23; SE = 2.20; *p*‐value = 0.02), the interaction between cyclone incidence and Month 1 (Cyclone year × Month 1: estimate = −10.77; SE = 5.28; *p*‐value = 0.04), and the interaction between distance from Lake Urema and Month 1 (Lake distance × Month 1: estimate = −0.59; SE = 0.25; *p*‐value = 0.02).

By the second month after the cyclone (Month 2), the spatial distribution of baboon detections had already renormalized, once again resembling the pattern seen before the cyclone and in non‐cyclone years of higher baboon detection rates at sites closer to Lake Urema (Figure 6). This trend persisted until the fifth through seventh months after Cyclone Idai made landfall, at which point baboon spatial distributions in the cyclone year appear to begin diverging slightly from non‐cyclone‐year distributions (Figure 6). Despite this slight trend, there were no further significant effects or interaction effects.

### 3.3 Relative Abundance Before and After Cyclone Idai

**FIGURE 7:**
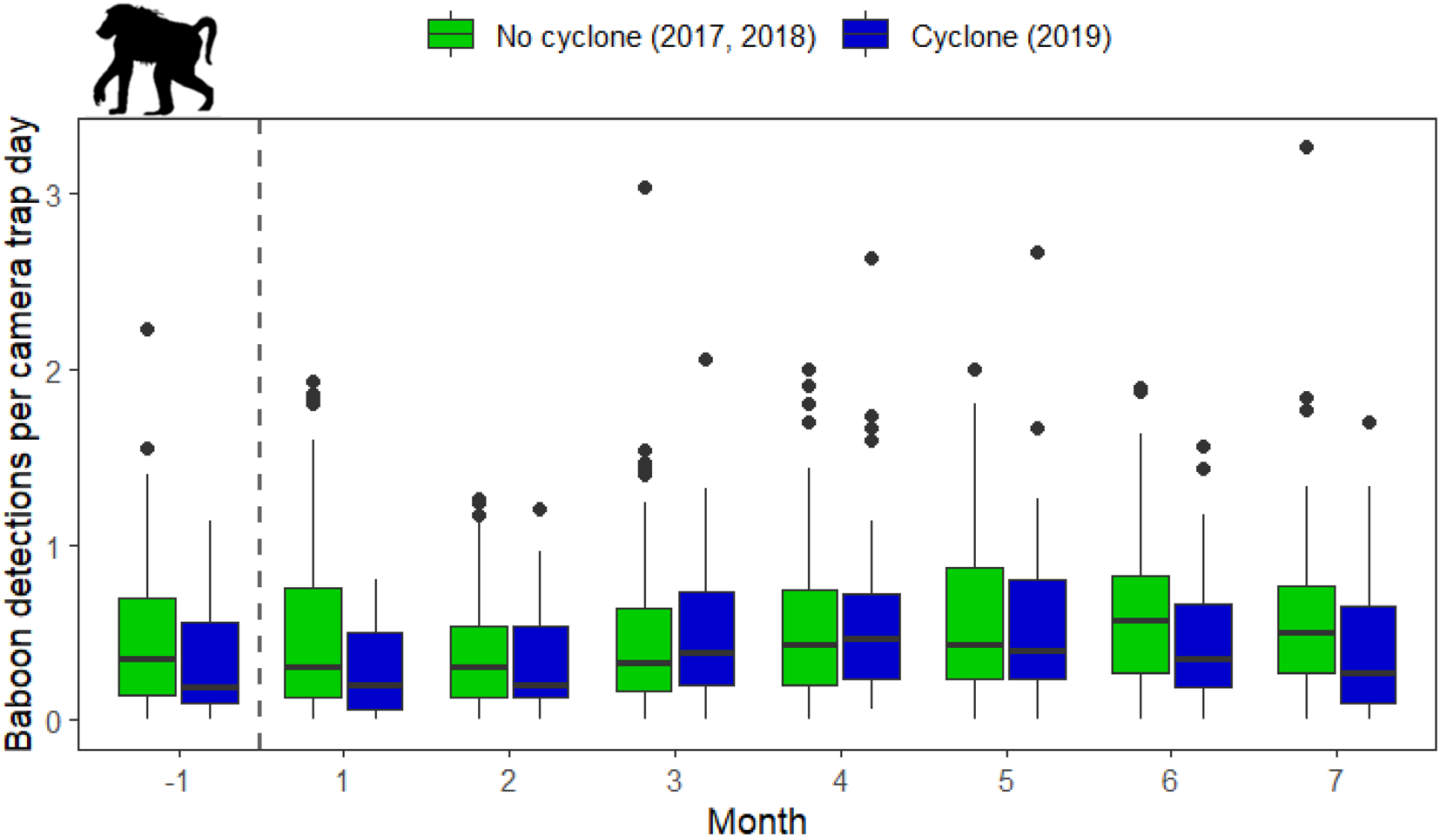
Differences in baboon detections per camera trap day (an index of relative abundance) across matched months in non‐cyclone years (2017 and 2018) and the year of Cyclone Idai (2019). The dashed vertical line represents the date when Cyclone Idai made landfall in Gorongosa National Park (March 15, 2019).

Baboon detections per camera trap day (an index of relative abundance) were largely comparable across matched months in the year of the cyclone and non‐cyclone years. The greatest difference was found in Month 1, with fewer baboon detections in the first month after the incidence of Cyclone Idai (mean = 0.28 ± 0.27) than in the matched time interval in the two prior years (mean = 0.56 ± 0.60). For the other matched months, mean baboon detections per camera trap day were more stable across cyclone and non‐cyclone years, with mean cyclone‐year detections even exceeding those observed in the non‐cyclone years in Months 3 and 4 (Figure 7).

**FIGURE 8:**
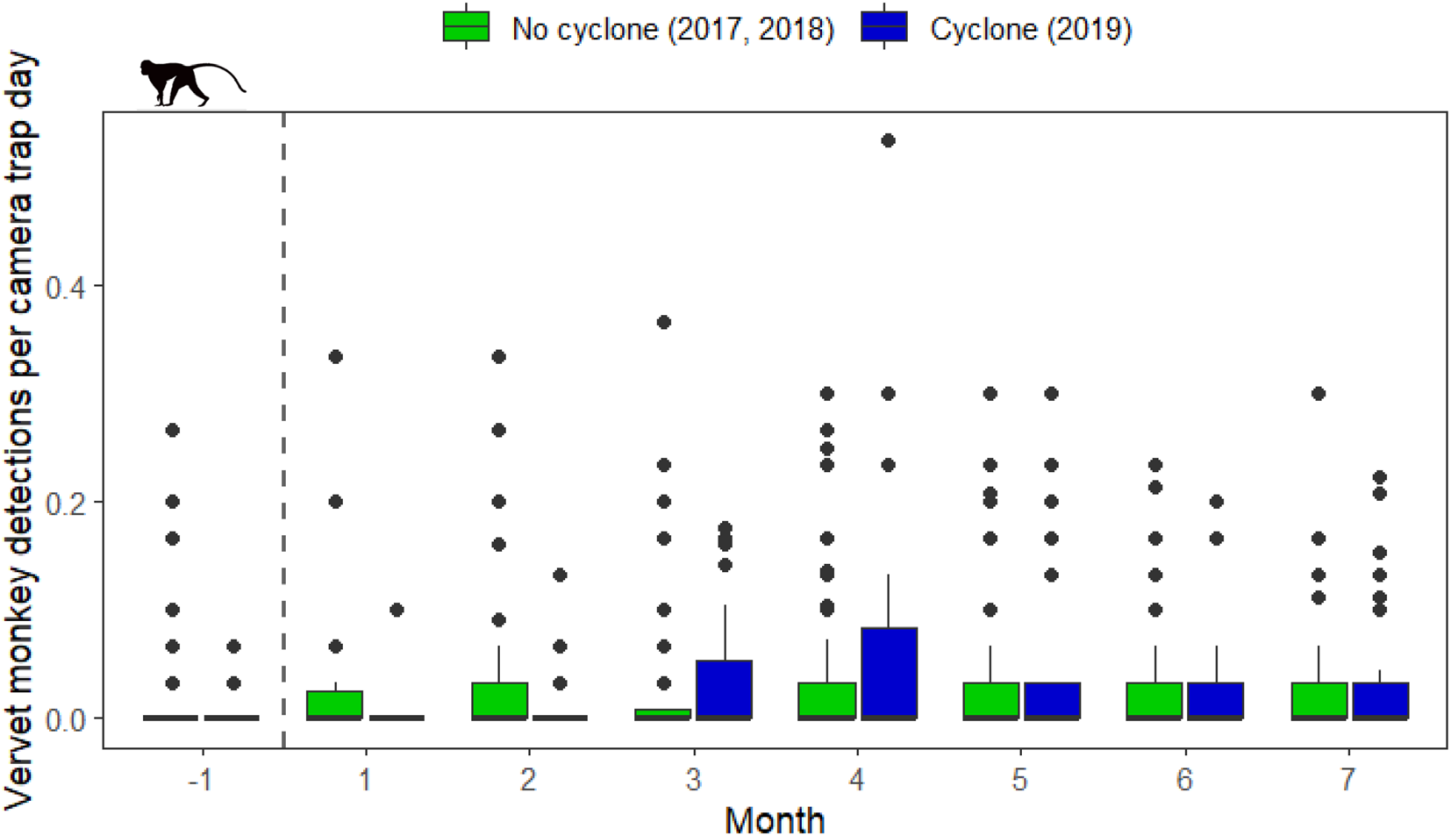
Differences in vervet monkey detections per camera trap day (an index of relative abundance) across matched months in non‐cyclone years (2017 and 2018) and the year of Cyclone Idai (2019). The dashed vertical line represents the date when Cyclone Idai made landfall in Gorongosa National Park (March 15, 2019).

For vervet monkeys, the greatest difference between cyclone‐year and non‐cyclone‐year detections per camera trap day in matched months occurred in Month 4, where average vervet monkey detections were higher in 2019 (mean = 0.06 ± 0.11) compared to 2017 and 2018 (mean = 0.03 ± 0.06). The second greatest matched difference was found in Month 1, with fewer vervet monkey detections in the year of the cyclone (mean = < 0.01 ± 0.02) than in the two prior years (mean = 0.03 ± 0.07). For the remaining matched months, mean vervet monkey detections per camera trap day were more stable across cyclone and non‐cyclone years, with mean cyclone‐year detections being lower than those observed in non‐cyclone years only during Months − 1, 2, and 6 (Figure 8). Across cyclone and non‐cyclone years, vervet monkey detections were low in month − 1 relative to other months. In this interval, detections during non‐cyclone years were at an all‐time low (mean = 0.02 ± 0.06), and we observed the second lowest recorded cyclone‐year detections (mean = < 0.01 ± 0.01), surpassed only by those recorded in the month immediately after Cyclone Idai.

## 4 Discussion

Our study demonstrates that Cyclone Idai and the associated severe flooding had a significant impact on the spatial distribution of baboons in Gorongosa National Park. Baboons that inhabited areas where flooding was most severe appear to have changed their spatial distributions, shifting their usual home ranges to occupy areas clear of the floodwater in the immediate aftermath of the cyclone. Following this initial disruption, baboon spatial distributions renormalized relatively quickly post‐cyclone, matching spatial distributions observed in the two prior years within 8 weeks of the cyclone. Baboon detections per camera trap day (an index of relative abundance) remained relatively stable across time, aside from an initial dip in the immediate aftermath of Cyclone Idai. This latter finding must be treated cautiously since the method used to determine the number of detections per camera trap day across each set of conditions, although a widely used index of relative abundance (O'Brien 2011; O'Brien, Kinnaird, and Wibisono 2003), does not account for potential variations in detectability over space and time (Sollmann et al. 2013). However, supporting this finding, systematic aerial wildlife counts conducted in the park did not detect a dramatic decrease in total baboon troops in the year following the cyclone (Stalmans, Peel, and Gonçalves 2018; Stalmans and Peel 2016, 2020). Thus, we can infer that baboons in Gorongosa National Park were largely able to adjust their ranging behavior to escape the rising floodwaters, providing them with a buffer against the immediate negative impacts of Cyclone Idai.

That vervet monkey detections were substantially lower than baboon detections across our study likely reflects both an actual occupancy difference and lower detectability due to the positioning of the cameras being better suited for surveying larger, ground‐dwelling animals than smaller, more arboreal species (Gaynor et al. 2021). Camera traps are known to be more effective at detecting animals with larger body sizes and greater home ranges, and more gregarious species that live in larger groups, as compared to smaller species with more compact home ranges and fewer individuals per group (Anile and Devillard 2016; Hofmeester, Rowcliffe, and Jansen 2017; Lyra‐Jorge et al. 2008; Randler and Kalb 2018; Rowcliffe et al. 2011; Tobler et al. 2008; Treves et al. 2010). Vervet monkeys are generally known to display a greater degree of arboreality than baboons, with the former being classified as semi‐terrestrial and the latter terrestrial (Estrada and Marshall 2024), meaning vervet monkeys may be less accurately monitored by terrestrial camera traps than baboons (as in Walton, Findlay, and Hill 2022).

While low detections prohibited us from analyzing whether vervet monkeys adjusted their ranging patterns in response to the cyclone, by the second month after the cyclone, their spatial distribution patterns were similar to non‐cyclone years, indicating they likely returned to their usual home ranges soon after the cyclone. Furthermore, following a decrease immediately after the cyclone, vervet monkey detection rates surpassed those of non‐cyclone years within 12 weeks, suggesting that vervet monkeys may have adopted behavioral adjustments such as flexible arboreality and ranging pattern shifts to evade the floodwaters. Interestingly, vervet monkey detection rates were low in both cyclone and noncyclone years during month − 1 relative to other months. Thus, the short‐lived detection decreases for vervet monkeys may represent variation in detection probability resulting from vervet monkeys shifting to a greater reliance on arboreality to evade floodwaters after Cyclone Idai and during Mozambique's wet season, rather than a true difference in abundance (Sollmann et al. 2013). As the present study used opportunistic data, future research using a combination of terrestrial and arboreal cameras (e.g., Bersacola et al. 2022) and behavioral observations would help to shed light on the true size of Gorongosa's vervet monkey population and their responses to ecological pressures.

Across mesoherbivores in Gorongosa National Park, Walker et al. (2023) found that smaller species were more vulnerable to the impact of Cyclone Idai, being unable to change their spatial distributions to the extent that would be required to escape the sudden flooding due to mobility constraints and exhibiting greater abundance declines. Of the species studied, it was the larger herbivores that were able to move to higher elevated areas clear of floodwater (Walker et al. 2023). A similar trend to our spatial distribution findings for baboons was observed for waterbuck ( *Kobus ellipsiprymnus* ; ~215 kg), warthog ( *Phacochoerus africanus* ; ~83 kg), impala ( *Aepyceros melampus* ; ~56 kg), and nyala ( *Tragelaphus angasii* ; ~98 kg) following Cyclone Idai (Walker et al. 2023). Bushbuck (*Tragelaphus sylvaticus*; ~43 kg), the smallest of the herbivores whose spatial distribution was analyzed using the camera trap survey, were the only studied species not to redistribute after the cyclone, with many individuals appearing to have perished (Walker et al. 2023).

Gray‐footed chacma baboons, while small‐bodied (~15–30 kg) relative to the species studied by Walker et al. (2023), appear to have been able to adjust their ranging patterns in response to the cyclone, which may be partially due to their advanced cognition, adaptation to both arboreal and terrestrial locomotion, high levels of dietary breadth, social tolerance, and evolved behavioral flexibility (Alberts and Altmann 2006; Barrett and Henzi 2008; Fischer et al. 2019; Jolly 1993; Sapolsky 2006). While the interpretations we can make for vervet monkeys are more limited due to the low number of detections across our study, the relative abundance patterns observed leave open the possibility that vervet monkeys (~4 kg) may also have been able to largely evade the Cyclone Idai floodwaters through behavioral adjustments. In common with baboons, vervet monkeys are known to be highly adaptable as a species, surviving in harsh conditions and responding flexibly to change (Herzog et al. 2016, 2020; Jaffe and Isbell 2009; McDougall et al. 2010; Pasternak et al. 2013; Wrangham 1981), such as adjusting their activity to spend more time resting at the expense of feeding to cope in extreme heat (McFarland et al. 2014) and exhibiting foraging flexibility in response to available food resources and the frequency of human interactions in urban environments (Thatcher, Downs, and Koyama 2020). Aside from our own species, baboons and vervet monkeys are the most widely distributed of the African primates, occupying a diverse range of habitats across the African continent (Wolfheim 1983). In line with our findings and interpretations, due to their high levels of adaptability, they are predicted to be less susceptible to extreme weather events than primates with lower adaptive capacity and higher sensitivity to ecological change (Zhang et al. 2019).

The evolution of high behavioral plasticity in primates is hypothesized to be linked to the high levels of ecological variability certain primate species have historically experienced across their ranges (Alberts and Altmann 2006)—the same variability that is suggested to have driven key aspects of human evolution (Potts 1998, 2013). In this regard, studies of extant primate responses to rapid climatic events, including those of highly adaptable species such as baboons and vervet monkeys, can provide useful insights into the ways that ecological pressures may have shaped primate and hominin evolution (Alberts and Altmann 2006; Fischer et al. 2019; King 2022).

Our study did not directly assess survival rates and cannot account for the possibility that some detections may correspond to immigrant primates moving into new areas, rather than the return of pre‐existing troops. Thus, our findings do not negate the possibility that Cyclone Idai may have caused primate populations to decline, even if the rate of detections remained relatively stable. Among the semi‐habituated floodplain troop of baboons in Gorongosa National Park which Lewis‐Bevan conducted focal follows of from 2018 to 2019, several infants and juveniles disappeared post‐cyclone, suggesting that younger individuals are more vulnerable to extreme weather events (Lewis‐Bevan et al. 2020), as has been observed among other primates. For example, following Hurricane Iris, infants and subadult male black howler monkeys ( *Alouatta pigra* ) in Belize experienced the highest mortality rates, possibly as a result of heightened social instability increasing contact with unfamiliar adult males and leading to elevated rates of infanticide (Pavelka et al. 2003). In addition, it is possible that experience may confer a survival advantage when dealing with extreme environmental conditions, with adults faring better than immature individuals due to acquired knowledge. Alternatively, or in combination with this, the stress caused by the incidence of extreme weather events and food shortages occurring in their aftermath may disrupt the lactation of mothers, compromising their ability to provide for their young. In line with this, studies of lemur species in Madagascar have shown that cyclones may be associated with reduced fecundity, negatively affecting birth rates and first‐year survival among Milne Edward's sifakas ( *Propithecus edwardsi* ) (Dunham, Erhart, and Wright 2011), and reducing food availability leading to nutritional stress and low numbers of births among lemurs inhabiting coastal forests (Ratsimbazafy 2002, 2007). Thus, while our results suggest that primates in Gorongosa National Park may have been less vulnerable to the impact of Cyclone Idai than other comparably sized mammals, we cannot assume that the population was unaffected, or that there will be no longer‐term consequences as a result of the cyclone's impact on food availability and reproductive fitness (Behie and Pavelka 2005; Johnson et al. 2011; Morcillo et al. 2020; Pavelka et al. 2003; Pavelka, McGoogan, and Steffens 2007; Schaffner et al. 2012; Testard et al. 2021; Watowich et al. 2022).

As in any natural ecosystem, abiotic and biotic pressures are dynamic in Gorongosa National Park. The resulting annual variability makes our comparison of detection data from the year of the cyclone to the two prior years admittedly imperfect in ways that cannot be controlled. While the incidence of Cyclone Idai presented an unexpected “natural experiment,” we highlight the need for long‐term data collection to assess how individuals and populations respond relative to long‐term averages. Future research should seek to increase our understanding of how these primates adjust their behavior to cope year‐on‐year with the highly seasonal environment of Gorongosa National Park, with its cycle of annual flooding followed by harsh droughts. Filling this gap in our knowledge will provide a more complete understanding of the primates of Gorongosa National Park, the selective forces shaping their behavior and evolution, and their ability to withstand future extreme ecological change.

## Author Contributions

**Megan Beardmore‐Herd:** conceptualization (lead), formal analysis (lead), funding acquisition (lead), investigation (lead), methodology (lead), writing – original draft (lead). **Meredith S. Palmer:** data curation (lead), formal analysis (equal), methodology (lead), resources (equal), software (equal), writing – review and editing (equal). **Kaitlyn M. Gaynor:** data curation (lead), formal analysis (supporting), resources (equal), software (equal), writing – review and editing (equal). **Susana Carvalho:** conceptualization (supporting), supervision (lead), writing – review and editing (equal).

## Conflicts of Interest

The authors declare no conflicts of interest.

## Supporting information


**Data S1.** Supporting Information.

## Data Availability

Data used in this study will be made available on Dryad if accepted for publication.
