# Supplementary material for: Effects of an Extreme Weather Event on Primate Populations
Source: Am J Biol Anthropol. 2025 Jan 6;186(1):e25049. doi: 10.1002/ajpa.25049 (PMC11701873; doi:10.1002/ajpa.25049)
Supplement: Supplementary file 1 — Data S1. Supporting Information. [file AJPA-186-e25049-s001.docx]

Supporting information – Effects of an Extreme Weather Event on Primate Populations

**Table 1: Descriptive statistics for baboon detections across the Gorongosa National Park camera trap grid.** Month (relative to Cyclone Idai) intervals correspond to the following periods of time: -1 = 13^th^ February – 14^th^ March; 1 = 16^th^ March – 14^th^ April; 2 = 15^th^ April – 14^th^ May; 3= 15^th^ May – 13^th^ June; 4 = 14^th^ June – 13^th^ July; 5 = 14^th^ July – 12^th^ August; 6 = 13^th^ August – 11^th^ September; 7 = 12^th^ September – 11^th^ October. The blue zigzagged horizontal line represents the date when Cyclone Idai made landfall in Gorongosa National Park (15^th^ March 2019).

| ***Month (relative to Cyclone Idai)*** | ***2017*** | | ***2018*** | | ***2019 (Cyclone year)*** | |
| --- | --- | --- | --- | --- | --- | --- |
|  | ***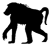 Total baboon detections*** | ***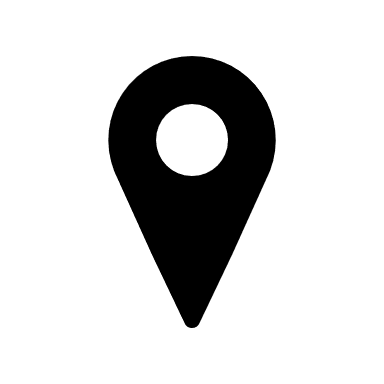 Total detection sites*** | 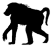 ***Total baboon detections*** | 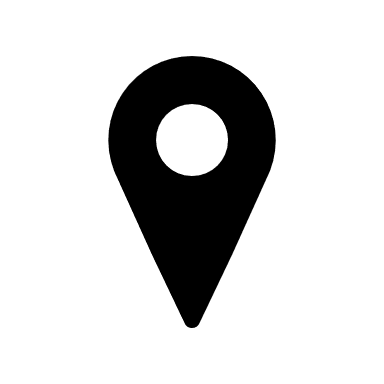 ***Total detection sites*** | 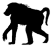 ***Total baboon detections*** | 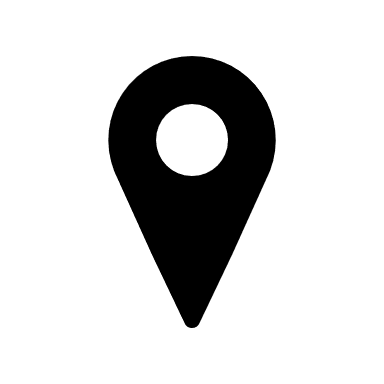 ***Total detection sites*** |
| ***-1*** | 402 | 27 | 260 | 17 | 275 | 24 |
| ***1*** | 458 | 25 | 313 | 17 | 214 | 21 |
| ***2*** | 385 | 31 | 224 | 25 | 265 | 24 |
| ***3*** | 481 | 37 | 456 | 33 | 457 | 40 |
| ***4*** | 817 | 51 | 528 | 41 | 694 | 38 |
| ***5*** | 917 | 44 | 638 | 40 | 698 | 40 |
| ***6*** | 894 | 44 | 598 | 38 | 603 | 37 |
| ***7*** | 874 | 40 | 559 | 37 | 438 | 32 |

**Table 2: Descriptive statistics for vervet monkey detections across the Gorongosa National Park camera trap grid.** Month (relative to Cyclone Idai) intervals correspond to the following periods of time: -1 = 13^th^ February – 14^th^ March; 1 = 16^th^ March – 14^th^ April; 2 = 15^th^ April – 14^th^ May; 3= 15^th^ May – 13^th^ June; 4 = 14^th^ June – 13^th^ July; 5 = 14^th^ July – 12^th^ August; 6 = 13^th^ August – 11^th^ September; 7 = 12^th^ September – 11^th^ October. The blue zigzagged horizontal line represents the date when Cyclone Idai made landfall in Gorongosa National Park (15^th^ March 2019).

| ***Month (relative to Cyclone Idai)*** | ***2017*** | | ***2018*** | | ***2019 (Cyclone year)*** | |
| --- | --- | --- | --- | --- | --- | --- |
|  | ***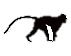 Total vervet detections*** | ***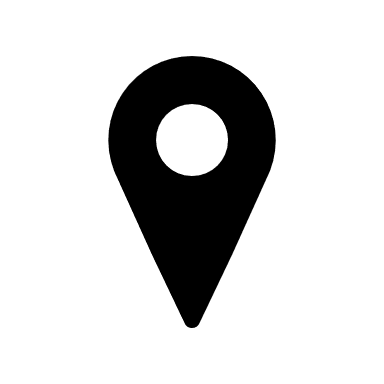 Total detection sites*** | 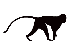 ***Total vervet detections*** | 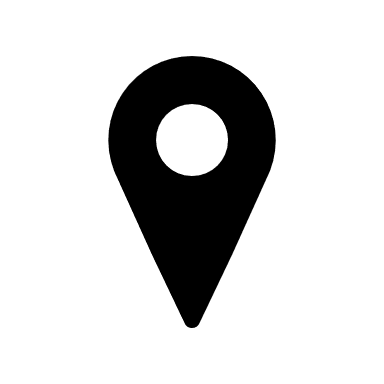 ***Total detection sites*** | 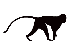 ***Total vervet detections*** | 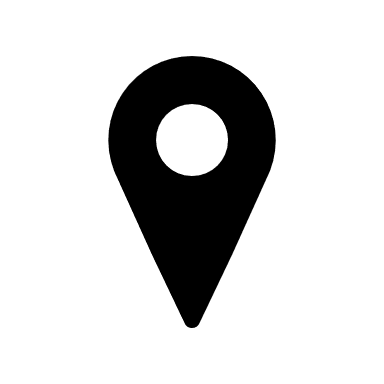 ***Total detection sites*** |
| ***-1*** | 31 | 7 | 3 | 2 | 4 | 3 |
| ***1*** | 30 | 7 | 6 | 5 | 3 | 1 |
| ***2*** | 23 | 6 | 19 | 8 | 8 | 4 |
| ***3*** | 24 | 8 | 28 | 10 | 31 | 16 |
| ***4*** | 57 | 17 | 25 | 12 | 69 | 18 |
| ***5*** | 52 | 17 | 25 | 10 | 50 | 13 |
| ***6*** | 41 | 17 | 33 | 11 | 34 | 15 |
| ***7*** | 31 | 15 | 29 | 11 | 28 | 11 |

**Table 3: Comparisons of the monthly distribution of baboon detections across the year of Cyclone Idai (2019) and two non-cyclone years (2017 and 2018).** The GLMM used predictors of ‘Month’ (a factorial variable with 8 levels representing the intervals corresponding to months since the cyclone made landfall, ranging from -1 to 7), ‘Cyclone incidence’ (a binary variable, where years were coded based on the incidence of Cyclone Idai), and ‘Lake distance’ (the log10-transformed distance in meters from Lake Urema). The reference conditions were Month -1 and Non-cyclone year. ‘SE’ denotes the standard error. Results have been rounded to two decimal places. Asterisks indicate statistical significance of P-values (* = < 0.05, ** = < 0.01, *** = < 0.001).

| **Baboon model fixed effects** | **Estimate** | **SE** | **Z value** | **P-value** |
| --- | --- | --- | --- | --- |
| (Intercept) | -3.73 | 2.00 | -1.87 | 0.06 |
| Cyclone year | 0.55 | 2.58 | 0.21 | 0.83 |
| Lake distance | -0.33 | 0.22 | -1.46 | 0.14 |
| **Month = 1** | **5.23** | **2.20** | **2.38** | **0.02*** |
| Month = 2 | 2.61 | 2.21 | 1.18 | 0.24 |
| Month = 3 | 0.30 | 2.13 | 0.14 | 0.89 |
| Month = 4 | -0.86 | 1.87 | -0.46 | 0.65 |
| Month = 5 | -1.37 | 1.87 | -0.73 | 0.46 |
| Month = 6 | -0.90 | 1.88 | -0.48 | 0.63 |
| Month = 7 | -0.64 | 1.90 | -0.33 | 0.74 |
| Cyclone year x Lake distance | -0.07 | 0.29 | -0.24 | 0.81 |
| **Cyclone year x Month 1** | **-10.77** | **5.28** | **-2.04** | **0.04*** |
| Cyclone year x Month 2 | 2.54 | 4.77 | 0.53 | 0.59 |
| Cyclone year x Month 3 | 1.09 | 3.64 | 0.30 | 0.77 |
| Cyclone year x Month 4 | 0.86 | 3.39 | 0.25 | 0.80 |
| Cyclone year x Month 5 | -3.07 | 3.14 | -0.98 | 0.33 |
| Cyclone year x Month 6 | -3.65 | 3.21 | -1.14 | 0.26 |
| Cyclone year x Month 7 | -6.20 | 3.40 | -1.83 | 0.07 |
| **Lake distance x Month 1** | **-0.59** | **0.25** | **-2.41** | **0.02*** |
| Lake distance x Month 2 | -0.31 | 0.25 | -1.24 | 0.22 |
| Lake distance x Month 3 | -0.07 | 0.24 | -0.30 | 0.76 |
| Lake distance x Month 4 | 0.03 | 0.21 | 0.14 | 0.89 |
| Lake distance x Month 5 | 0.09 | 0.21 | 0.42 | 0.67 |
| Lake distance x Month 6 | 0.04 | 0.21 | 0.20 | 0.85 |
| Lake distance x Month 7 | 0.01 | 0.21 | 0.07 | 0.95 |
| **Cyclone year x Lake distance x Month 1** | **1.25** | **0.58** | **0.03** | **0.03*** |
| Cyclone year x Lake distance x Month 2 | -0.24 | 0.53 | -0.46 | 0.65 |
| Cyclone year x Lake distance x Month 3 | -0.09 | 0.40 | -0.23 | 0.82 |
| Cyclone year x Lake distance x Month 4 | -0.07 | 0.38 | -0.20 | 0.84 |
| Cyclone year x Lake distance x Month 5 | 0.35 | 0.35 | 0.99 | 0.32 |
| Cyclone year x Lake distance x Month 6 | 0.42 | 0.36 | 1.17 | 0.24 |
| Cyclone year x Lake distance x Month 7 | 0.73 | 0.38 | 1.91 | 0.06 |
